# Empathy bodyssence: temporal dynamics of sensorimotor and physiological responses and the subjective experience in synchrony with the other’s suffering

**DOI:** 10.3389/fpsyg.2024.1362064

**Published:** 2024-03-21

**Authors:** Alejandro Troncoso, Kevin Blanco, Álvaro Rivera-Rei, David Martínez-Pernía

**Affiliations:** ^1^Center for Social and Cognitive Neuroscience, School of Psychology, Adolfo Ibáñez University, Santiago, Chile; ^2^Geroscience Center for Health and Brain Metabolism (GERO), Santiago, Chile

**Keywords:** bodyssence, empathy, enaction, neurophenomenology, empirical 5E approach, first-person view, phenomenology, mixed-method study

## Abstract

**Background:**

Empathy is foundational in our intersubjective interactions, connecting with others across bodily, emotional, and cognitive dimensions. Previous evidence suggests that observing individuals in painful situations elicits whole bodily responses, unveiling the interdependence of the body and empathy. Although the role of the body has been extensively described, the temporal structure of bodily responses and its association with the comprehension of subjective experiences remain unclear.

**Objective:**

Building upon the enactive approach, our study introduces and examines “bodyssence,” a neologism formed from “body” and “essence.” Our primary goal is to analyze the temporal dynamics, physiological, and phenomenological elements in synchrony with the experiences of sportspersons suffering physical accidents.

**Methods:**

Using the empirical 5E approach, a refinement of Varela’s neurophenomenological program, we integrated both objective third-person measurements (postural sway, electrodermal response, and heart rate) and first-person descriptions (phenomenological data). Thirty-five participants watched videos of sportspersons experiencing physical accidents during extreme sports practice, as well as neutral videos, while standing on a force platform and wearing electrodermal and heart electrodes. Subsequently, micro-phenomenological interviews were conducted.

**Results:**

Bodyssence is composed of three distinct temporal dynamics. Forefeel marks the commencement phase, encapsulating the body’s pre-reflective consciousness as participants anticipate impending physical accidents involving extreme sportspersons, manifested through minimal postural movement and high heart rate. Fullfeel, capturing the zenith of empathetic engagement, is defined by profound negative emotions, and significant bodily and kinesthetic sensations, with this stage notably featuring an increase in postural movement alongside a reduction in heart rate. In the Reliefeel phase, participants report a decrease in emotional intensity, feeling a sense of relief, as their postural control starts to reach a state of equilibrium, and heart rate remaining low. Throughout these phases, the level of electrodermal activity consistently remains high.

**Conclusion:**

This study through an enactive approach elucidates the temporal attunement of bodily experience to the pain experienced by others. The integration of both first and third-person perspectives through an empirical 5E approach reveals the intricate nature of bodyssence, offering an innovative approach to understanding the dynamic nature of empathy.

## 1 Introduction

Empathy is a critical component of our intersubjective life, playing a vital role in our ability to connect with others on a bodily, emotional, and cognitive level. It refers to our basic capacity to share, feel, or recognize another person’s world (Eklund and Meranius, 2021). This ability to resonate with others involves not only psychological processes but also whole-body mechanisms that enable us to experience the emotions of another person firsthand (Gallese, 2014; de Waal and Preston, 2017; Riečanský and Lamm, 2019). Additionally, these mechanisms allow us to respond to another’s emotions through action (Gallese, 2014; de Waal and Preston, 2017; Riečanský and Lamm, 2019). This cycle of perception and action through the body has been a prominent focus in the field over the past few decades. Studies examining the role of the body have investigated movement responses, autonomic reactions, and the activation of various areas of the motor control system in response to images or videos of another person in pain [for reviews, see Riečanský and Lamm, 2019 and Troncoso et al., 2023].

For instance, physiological and motor changes have been reported during the observation of others’ emotions (Hein et al., 2011; Gea et al., 2014; Lelard et al., 2014; Mouras and Lelard, 2021). Another observation found that viewing faces in pain leads to increased amplitude of body sway in subjects and correlates with higher empathic subjective scores, suggesting that changes in postural control may be associated with approach and cooperative responses (Gea et al., 2014; Lebert et al., 2020). Concerning cardiac activity, individuals’ emotional regulation of empathy through up-regulation and down-regulation while watching emotional videos were linked to increased and decreased subjective scores of situational empathy and distinct changes in heart rate variability compared to individuals in the control condition (No-regulation) while participants viewed emotional videos (Jauniaux et al., 2020). Complementing these observations, studies employing neuroimaging techniques have demonstrated that these bodily responses are reflected in brain activity. Indeed, numerous neuroimaging studies have indicated that activity in the somatosensory and motor cortices, typically associated with first-hand pain, is also triggered in response to the pain of others (Bufalari et al., 2007; Lamm et al., 2007; Riečanský and Lamm, 2019). These activations occur at multiple levels of the nervous system, from the cerebral cortex down to the spinal cord (Riečanský and Lamm, 2019). These findings underscore the intricate interplay between the brain and body in empathic experiences, offering a tangible illustration of how the body is fundamental in the empathic experience.

While the study of the role of the body in empathy research has furnished valuable insights and heavily influenced empirical empathy research, there is an escalating need for a more integrated and holistic approach. Current methodologies, largely centered on correlating neurophysiological and subjective data (self-report questionnaires), may not fully capture the intrinsic dynamics of empathy within lived and situational contexts (Varela et al., 1991; Gallagher, 2003; De Jaegher and Di Paolo, 2007; Olivares et al., 2015). This is where the enactive approach, prioritizing the phenomenological experience and the mutual co-constitution of agent and environment, offers a unique and complementary lens for understanding empathy (Varela et al., 1991; Thompson, 2007; Colombetti, 2014; Newen et al., 2018; Troncoso et al., 2023). This approach proposes that the primary source of experience and understanding of others is through and with the body (Gallagher, 2012; Tanaka, 2015; Fuchs, 2017). Through our lived body we experience the physical expressions of others as meaningful actions that communicate their intentions, needs, and objectives within a shared context (Gallagher, 2012; Tanaka, 2015; Fuchs, 2017). In this sense, phenomenological descriptions have demonstrated the multidimensional and complex dynamic nature of subjective experiences of empathy and how they are formed by neurophysiological responses (Grice-Jackson et al., 2017a; Martínez-Pernía et al., 2023). For example, Grice-Jackson et al. (2017b) used bodily experience descriptions to distinguish different responders in a classic empathy for pain study. This phenomenological clustering revealed different functional brain connectivity between different kinds of conscious bodily feeling (Grice-Jackson et al., 2017b).

Building upon this foundational understanding, it is crucial to address the temporal dynamics of empathy. The enactive perspective posits that empathy is not just an individual experience but a collaborative process involving mutual interaction in a shared environment (Colombetti, 2014; Laroche et al., 2014; Fuchs, 2017). Empathy, therefore, is not a static response but a temporal dance of mutual adaptation where our reactions harmonize with the actions, emotions, and postures of those around us (Fuchs, 2013, 2017). Such dynamic interactions influence both parties involved as highlighted by studies showing spontaneous physiological coordination between individuals simply due to their co-presence (Golland et al., 2015). Emphasizing these temporal dynamics is paramount not only for a deeper understanding of the neurological facets of empathy but also for developing empathy theories (Golland et al., 2015).

In summary, the enactive approach offers a fresh and integrative perspective on empathy, highlighting the intricate interplay of embodiment, phenomenological insights, and their timely resonance with the environment. Despite the advancements in the enactive approach, the ever-evolving discipline of empathy research requires the ongoing development and refinement of concepts and more empirical evidence to support its theoretical formulations. Contemporary literature is replete with concepts such as the “lived body,” “affordance,” and “bodily resonance” seeking a more intricate and holistic understanding of the human being. Yet, there remains a significant empirical gap in understanding how these biological, subjective, and temporal processes converge. Seeking to bridge this chasm, we introduce the notion of “bodyssence.” This neologism composed of the words “body” and “essence,” represents a holistic exploration that bridges our neurophysiological responses with our subjective experiences. It underscores the dynamic interplay of these dimensions, both in the moment’s immediacy and over time. This concept seeks to understand how our embodied experiences dynamically evolve and resonate with both our internal and external states in synchrony with the environment.

Therefore, in this research, our objective is to explore “bodyssence” more deeply within the context of empathy and to examine how the temporal evolution of motor, physiological, and phenomenological correlates, in synchrony with other, shapes our empathetic consciousness. Thus, we employed a refinement of Varela’s neurophenomenological program (Varela, 1996) termed the empirical 5E approach (Troncoso et al., 2023), in which data from the sensorimotor system and physiological responses are collected using a mobile brain–body imaging system (MoBi) alongside the subjective experience. Participants were exposed to videos of people in physical accident situations (empathy for pain condition) and neutral videos that allowed the assessment of a baseline response from the participants (baseline condition). In the exposure, postural and physiological responses (electrocardiogram and electrodermal activity) were examined. Next, a micro-phenomenological interview was performed to explore the multi-layered dimensions (bodily sensations, emotions, and motivations) and temporal aspects of empathic experience. Ultimately, the integration of motor and physiological data will capture a multifaceted interplay of temporal, corporeal, and dynamic shifts as they relate to the subject of empathy, culminating in a comprehensive view of empathy for pain through our ‘bodyssence’ conceptualization. We hypothesize that a subjective climax experienced during the fall of sportspersons may be associated with an increase in anteroposterior movement, electrodermal activity, and heart rate. Additionally, following the peak climax, we hypothesize that all variables will decrease compared to the highest point, while the subjective experience will exhibit a decrease of anguish.

With the introduction of the term “bodyssence,” we seek to clarify this intertwined terrain of empathy. “Bodyssence” not only offers a more detailed and comprehensive perspective on the interweaving of bodily, subjective processes in interaction with the environment but also establishes a clear research methodology that transitions from an enactive theoretical framework to a precise scientific mixed-method study (empirical 5E approach). With this initiative, we aim to energize the scientific community to embark on empirical research within the enactive perspective, especially since the majority of this field has remained theoretical. The scientific validity of this paradigm demands, and indeed compels both philosophers and neuroscientists alike, and associated disciplines, to uncover scientific findings that will make this vision enduring.

## 2 Methods

### 2.1 Participants

From September 2017 to January 2018, thirty-five adults participated in the study (19 female; mean age 30.18 ± 6.62 years; range 21–47 years, mean years of education = 17.17 ± 2.35)^1^, all of whom were Latin American and Spanish-speaking. They were all healthy and did not have any cognitive or physical conditions that could affect their normal psychological and motor faculties. To corroborate that participants met the inclusion criteria, brief interview questionnaires were administered using the Montreal Cognitive Assessment (MoCA; Nasreddine et al., 2005), the Beck’s Depression Inventory (BDI-II, Beck et al., 1996), and State Trait Anxiety Inventory (STAI; Spielberger et al., 1970). The results of the questionnaires were as follows: MoCA = 28.57 ± 1.46; BDI-II = 5.4 ± 5.87; STAI = 49.4 ± 12.04.

All the participants signed informed consent. The study procedure was according to the Declaration of Helsinki principles. It was approved by the *“Scientific Ethics Committee of the Servicio de Salud Metropolitano Oriente”* and the *“Research in Humans Being Ethics Committee of the Medicine Faculty, Universidad de Chile.”*

### 2.2 Construction and validation of the emotional stimuli

For the construction of the empathy for pain and baseline video conditions, 12 scenes were produced for each condition using audiovisual material found under Creative Commons licenses on the web. Each scene had an average duration of 7–11 s. The scenes for the pain condition included images of sportspersons suffering intense physical accidents during the practice of extreme sports (e.g., parkour, high mountain slackline, acrobatic snowboarding). Scenes of dismemberment, disfigurement, or death were not used. Stimuli with significant camera movements or vibrations were also excluded, as were scenes that produced saccadic eye movements. All scenes for the empathy for pain condition had a similar sequence: a sportsperson skillfully practices a sports activity (pre-fall); next, the sportsperson starts losing balance until they have a strong impact with the ground (fall); and finally, the sportsperson is seen moving after the impact (post-fall). In contrast, the baseline condition, consisting of images of domestic spaces, was established to gauge a fundamental neurophysiological response. This provides a foundational reference against which the heightened responses from the empathy for pain condition can be effectively compared over time.

After the empathy for pain and baseline were constructed, they were validated with 65 university students (38 female and 27 males; average age = 19.34 ± 1.56) using the Self-Assessment Manikin scale (Bradley and Lang, 1994), which evaluates valence, arousal, and dominance on a 9-point Likert scale. Higher scores indicate pleasant valence, greater arousal, and dominance of the situation; lower scores indicate unpleasant valence, less arousal, and dominance of control over the situation. Finally, two 60-s videos were constructed (empathy for pain condition and baseline condition) each containing seven scenes. Paired t-tests showed that the videos selected for the empathy for pain stimuli were assigned significantly lower valence (pain: mean = 3.77 ± 1.94; baseline: mean = 4.97 ± 2.39; *t* (64) = −7.24, *p* < 0.001), higher arousal (pain: mean = 6.40 ± 1.78; baseline: mean = 3.02 ± 2.24; *t* (64) = 24.91, *p* < 0.001), and lower dominance (pain: mean = 5.31 ± 2.68; baseline: mean = 7.66 ± 2.25; *t* (64) = −14.59, *p* < 0.001) than baseline stimuli. The empathy for pain video was previously constructed and validated by our group, and has been utilized in prior works (Martínez-Pernía et al., 2020, 2023; Pizarro et al., 2023).

### 2.3 Procedure

Participants were asked to maintain a quiet stance while standing on a force plate with hip-width feet positioned, arms rested alongside their body, and at a 1-meter distance from a 40-inch screen TV. Each condition video (empathy for pain and baseline) was randomly reproduced on the screen while the postural control data and physiological data were collected. The postural control data were collected by a Bertec FP4060-05-PT brand stabilometric platform (Bertec Corporation, Columbus, Ohio, USA). A BIOPAC MP150 data acquisition and analysis system with AcqKnowledge software (BIOPAC Systems, Inc.) was used for the integration of all stabilometry signals The electrocardiogram (ECG) was recorded using disposable snap ECG electrodes in a modified lead II configuration, with one electrode positioned beneath the right clavicle and the other near the lower ribs on the left side. Concurrently, the electrodermal activity (EDA) was measured using disposable snap electrodes attached to the palmar surface of the distal phalanges of the first and second fingers.

A script made in MATLAB (MathWorks, Natick, MA, United States) was used to present the stimulus on a screen (40 inches) and send signals to the AcqKnowledge software for synchronization of the stimulus presentation and the stabilometry and physiological data. The ECG and the EDA were also collected and synchronized with the stimulus presentation through the BIOPAC. Immediately after participants finished watching each video, the 9-point Likert scale of the Self-Assessment Manikin was administered to explore intensity and arousal. After, a researcher conducted a phenomenological interview focused on the pain condition.

### 2.4 The phenomenological interview

All interviews were conducted in Spanish by the same researcher, recorded using an audio device, and subsequently transcribed verbatim. At the beginning of each interview, participants were asked to describe the videos they found unpleasant and then select the one that represented the most intense overall experience. Next, we asked about the overall temporality of all videos to corroborate a similar pattern among the unpleasant videos. This approach was employed to facilitate a clearer recollection of the experience. Interviews were conducted following the micro-phenomenological interview (Petitmengin, 2006).

The interviews were focused on multidimensional experiences (bodily, affective, sensory, attentional, etc.) at specific moments, as well as on the fluctuations of these dimensions throughout the experience. A vital aspect of the interviews was maintaining a continuous awareness (by both the researcher and the participant) of the suspension of the “natural attitude,” and judgmental stance toward one’s own experience (Hamilton et al., 2019).

Initially, the interview commenced with a description of the interview objectives and the embodied approach to questions and answers, concentrating on the experiences related to the video. Next, the participant was prompted to evoke the video experience. This evocation principle was crucial to elicit the participants’ pre-reflective descriptions and to vividly explore their past experiences (Petitmengin et al., 2018). The interview was developed by focusing on the synchronic aspect of the experience (e.g., “How do you feel about the video?” or “What is the feeling of tension like?”) and the diachronic dimensions (e.g., “After the feeling of tension, what happens?” or “At what point do you feel the tension?”). Another characteristic of the interview procedure was to recapitulate the participant’s responses to facilitate their recall.

### 2.5 Data analysis

#### 2.5.1 Behavioral and physiological data analysis

The stabilometric force (Fx, Fy, Fz) and moment (Mx, My, Mz) components were collected at a sample rate of 125 Hz. The center-of-pressure (COP) was computed for the anteroposterior and mid-lateral directions. The COP series were filtered with an 8 Hz fourth-order lowpass Butterworth filter. The EDA data was sampled at 500 Hz. The phasic component was estimated using the convex optimization approach (Greco et al., 2016). The statistical analysis was performed on the mean phasic component of the EDA (mS). The ECG data was sampled at 500 Hz. The R peaks were identified in MATLAB with the peak finder function. Each variable was analyzed through seven temporal windows of 1 s. The selection of these windows began by identifying the moment of the athlete’s fall. This event was identified as time 0. Next, three windows were taken before the athlete’s fall and another four windows after the fall for 1 s each. The seven windows of the baseline condition were selected by taking the central temporal part of each scene. Subsequently, and in each window, the anteroposterior (AP) amplitude of the CoP was calculated. The AP amplitude was chosen based on the sensitivity demonstrated in previous studies that investigated postural responses in emotional research (Lelard et al., 2019). For the EDA and ECG, the phasic component and heart rhythm were analyzed, respectively.

#### 2.5.2 First-person data analysis

**Figure 1:**
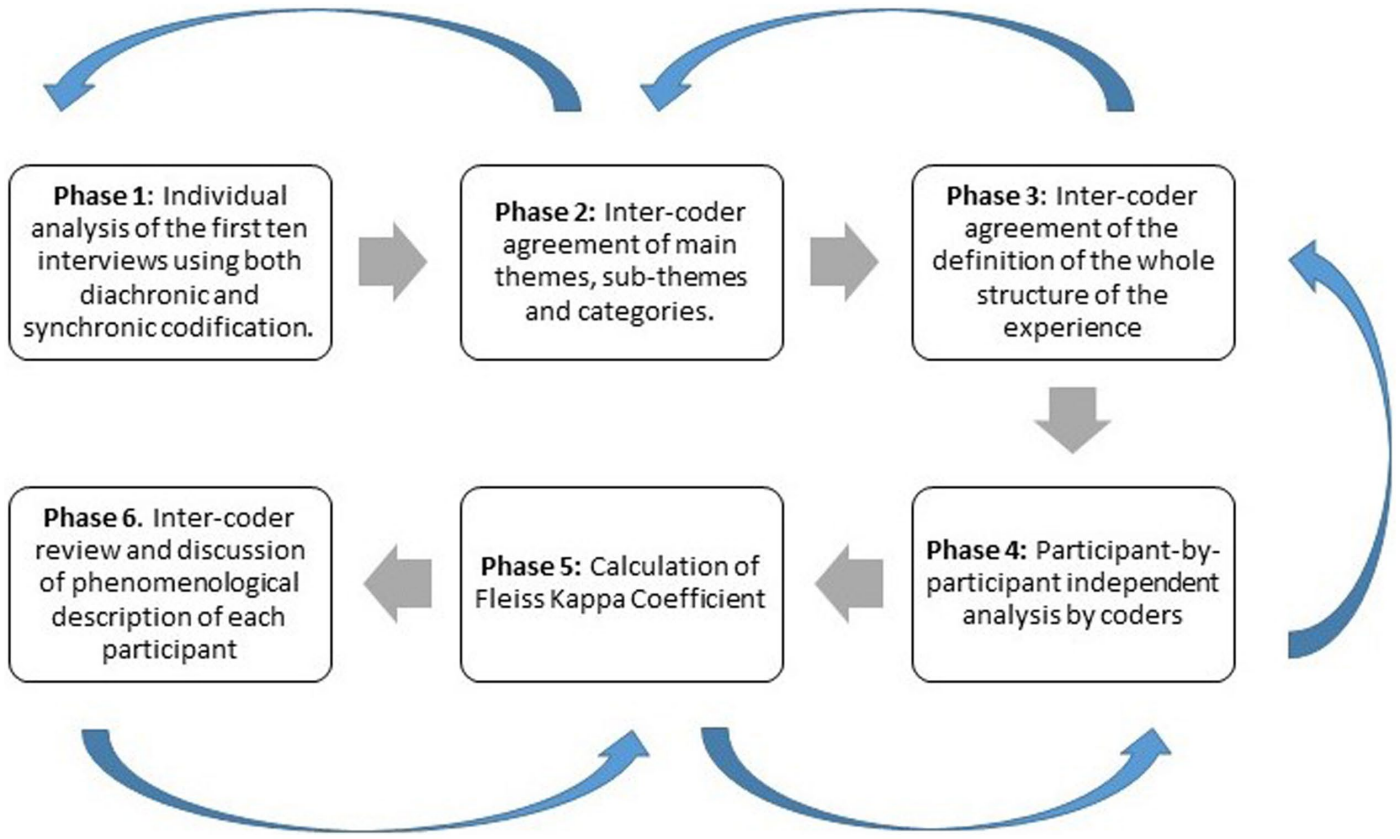
Intersubjective validation analysis. The blue arrows show the iterative process. The analysis starts with an independent codification of a part of the total research sample by each researcher. Phases 2 and 3 show the agreement of the formation and grouping of phenomenological categories. Once the emerging categories have been defined in Phase 4, the sample from Phase 1 and the total sample are analyzed again. If new categories appear, processes 2 and 3 are repeated (iterative process). In phase 5, the agreement is calculated using Fleiss’ Kappa. In phase 6, the experience structure and categories of each subject are reviewed among researchers. Only discrepancies between researchers and their sources are discussed. In case of doubts in the categories (definition and grouping), we return to the previous processes and reanalyze the subject.

To conduct the phenomenological analysis of the data, we used the descriptive phenomenological psychological method, hereafter, Giorgi’s method (Giorgi et al., 2017). This method centers the analysis on the meaning of the experience and aims to describe its structure by identifying central themes (Giorgi et al., 2017). In this sense, the psychological structure of the experience refers to how the subject makes sense of their own lived experience in the world. Further complementing this, we analyzed a diachronic structure, aligning the phenomenological categories with each temporal phase in line with the recommendations from the microphenomenological method (Valenzuela-Moguillansky and Vásquez-Rosati, 2019). To enhance the integrity and consistency of our phenomenological exploration, we adopted an iterative method within the triangulation process, including an inter-rater agreement index (Martínez-Pernía et al., 2023). The details of this comprehensive process will be outlined below (Figure 1).

Initially, and supported by Atlas.ti 9 qualitative data analysis software, each of the three researchers involved in the analysis (DMP, AT, KB) began with a thorough reading of the interview and extracting a concise overview to capture the essence of each participant’s entire described situation. After this initial step, the researcher underscored statements directly reflecting the experience and termed them ‘meaning units’. Throughout this process, the researchers transformed participants’ expressions into diachronic (elements of the experience evolving) and synchronic (elements of the experience occurring at a specific moment) codes that emphasized their inherent psychological significance. These codes were then specifically defined based on the first ten interviews (phase 1).

Following the initial coding, the researchers engaged in a process of abstraction, refining the detailed phenomenological codes such as “body tension” and “body anguish” into more abstract categories like “body feeling.” This was done by identifying shared experiences across participants based on the first ten interviews (phase 2). As primary themes (e.g., “bodily resonance”) and their associated subthemes (e.g., “affective quality”) emerged, the research team discussed them thoroughly. Through these deliberations, they achieved a unified understanding of the main themes, subthemes, categories, and temporal phases, thus ensuring the reliability and validity of the findings.

The next step (phase 3) involved capturing and describing the overall structure of the experience based on the analysis conducted in the first ten interviews. Because the themes provide insights into specific aspects of the experience, we integrated them to form a comprehensive structure. This structure progressed from particular elements to the participants’ fundamental comprehension, which was achieved by examining and systematically varying these specific elements to uncover their essence. While the initial abstraction process was conducted based on the first ten interviews until saturation was achieved, the refinement process continued throughout all subsequent interviews and phases.

Next, in phase 4, the researchers conducted, independently, a subject-by-subject analysis of all samples using the main themes, sub-themes, and categories labeled in phase 3. Subsequently, in phases 5 and 6, a novel inter-coder triangulation approach was employed to address potential errors, omissions, doubts, and disagreements among the researchers.

Phase 5 entailed calculating the Fleiss Kappa coefficient. The coefficients were used throughout the analysis process to assess the level of agreement between the researchers. By calculating the level of agreement, the researchers could identify errors, omissions, and disagreements. The average of the Kappa coefficient was 0.92 (for more information see https://osf.io/dtcr2/). This value indicates a high level of consensus among coders. The feedback provided in Phase 5 further aided in pinpointing and resolving any discrepancies among the researchers in Phase 6.

In the final step (phase 6), the researchers engaged in a collaborative review and discussion of the experiential dimensions of each analyzed participant based on the level of agreement with phase 5. This collaborative process allowed them to collectively examine and resolve any uncertainties or discrepancies related to definitions, errors, or omissions. Through this rigorous exchange of ideas, a consensus was reached among the researchers, thus enhancing the overall robustness and credibility of the study.

Our analysis included a process of iteration throughout the procedure. If during an interview analysis, a new theme or subtheme appeared or was modified, we reviewed all the previously analyzed data to maintain consistency among the new and previous categories. This review procedure and consistency were also implemented in the last stages.

The triangulation was performed in R statistical programming language to identify the phenomenological categories in which there was agreement or disagreement between the researchers.

### 2.6 Statistical analysis

Repeated-measures ANOVA with the two within-subjects factors (time windows and condition.) was used for each variable (postural movement, electrodermal activity and heart rate). Greenhouse–Geisser corrections were used when the assumption of homogeneity of covariances was violated (as determined by Mauchly tests of sphericity). Bonferroni-corrected *post hoc* pairwise comparisons were computed to examine interactions and omnibus main effects. All the analyses were performed using R Studio. A significance level of *p* = 0.05 was used for all statistical analyses.

All the data analyses of this study are included in the following link (https://osf.io/dtcr2/).

## 3 Results

The experimental manipulation successfully demonstrated differences between the visualization of videos that depict situations triggering empathy for pain (videos related to falls of sportspeople) and the baseline condition (neutral videos related to domestic spaces). This was indicated by significantly higher arousal levels in the pain condition (mean = 6.6 ± 1.33) compared to the baseline condition (mean = 2.46 ± 1.17, *p* < 0.001), along with notably lower valence in the pain condition (mean = 3.8 ± 2.07) in contrast to the baseline condition (mean = 6.4 ± 1.99, p < 0.001). In terms of result presentation, we will first detail the findings concerning sensorimotor and physiological responses, referred to as the third-person results (For a detailed examination of the data refer to open data https://osf.io/dtcr2/). Subsequently, we will delve into the phenomenological outcomes or the first-person results. Finally, we will provide an integrated view combining both the third-person and first-person findings, referred to as the “bodyssence result.”

### 3.1 Third-person results

#### 3.1.1 Postural movement

**Figure 2:**
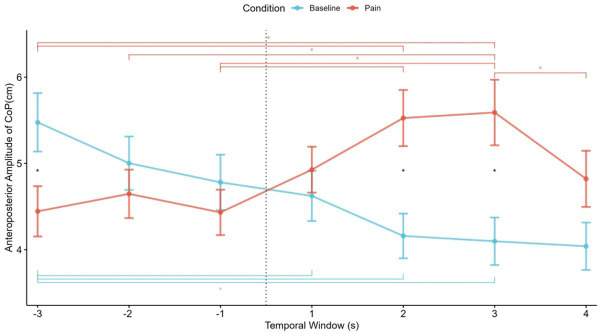
Temporal fluctuations of mean anteroposterior postural movement responses in participants during the visualization of pain-related videos (empathy for pain condition) and neutral videos. The dashed line between [−1] and [1] temporal window represents the fall of the sportsperson. The asterisk (*) denotes significant differences between conditions (black) and between temporal windows in empathy for pain (red) and baseline (cyan).

To compare the second-to-second temporal fluctuations in participants’ postural movement responses between the visualization of videos related to pain (empathy for pain condition) and neutral videos (baseline condition), an ANOVA with the factors Condition (2) and Time Window (7) was conducted. The ANOVA showed a significant interaction (*F*_6,204_ = 13.25, *p* < 0.001) between emotional conditions and the time window in the anteroposterior postural movement. The main effect of time windows was not statistically significant (*F*_6,204_ = 1.94, *p* = 0.076). There was a significant main effect of condition (*F*_6,204_ = 18.32, *p* < 0.001). As shown in Figure 2, there were significant differences in the time windows [−3], [2], and [3] (*p* < 0.05) between conditions. In the empathy for pain condition, there was a significant increase (*p* < 0.05) in the post-fall time windows when compared to the initial windows in specific time windows (for details see Figure 2). Interestingly, the last post-fall windows show significantly less postural movement than the previous one. In the baseline condition, there was a statistically significant decrease (*p* < 0.05) when comparing the initial windows to the final windows.

#### 3.1.2 Electrodermal activity

**Figure 3:**
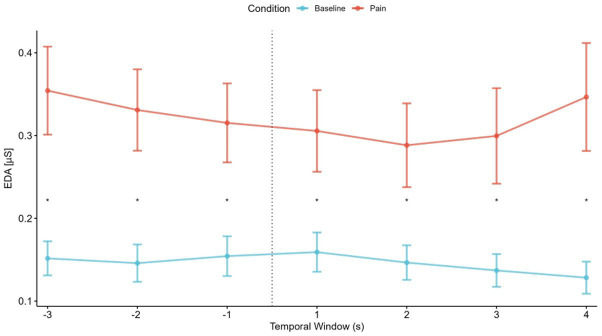
Temporal fluctuation in participants’ electrodermal activity responses to pain-related videos (empathy for pain condition) and neutral videos. The figure shows the temporal fluctuation of mean electrodermal activity. The dashed line between [−1] and [1] temporal window represents the fall of the sport-person. The asterisk (*) denotes significant differences between conditions (black).

To compare the temporal fluctuations in electrodermal activity between the empathy for pain condition and the baseline condition, an ANOVA with the factors Condition (2) and Time Window (7) was performed. The ANOVA showed a significant interaction (*F*_6,204_ = 3.04, *p* = 0.007) between emotional conditions and the time window in electrodermal activity. There was a significant main effect of time windows (*F*_6,204_ = 2.6, *p* = 0.019) and a significant main effect of condition (*F*_5,204_ = 15.24, *p* < 0.001). As shown in Figure 3, the empathy for pain condition reported significantly more phasic activity than the baseline condition in each temporal window (*p* ≤ 0.01). No significant differences were found in the pairwise comparisons between temporal windows at each condition.

#### 3.1.3 Heart rate

**Figure 4:**
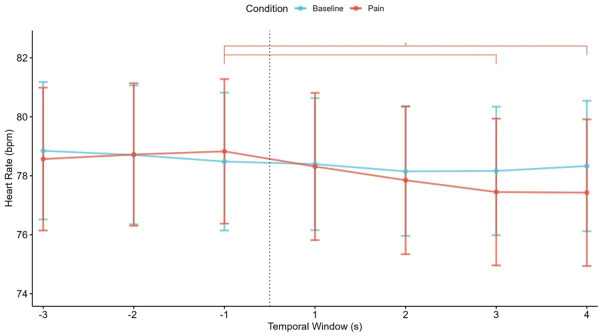
Temporal fluctuations in participants’ heart rate responses to pain-related videos (empathy for pain condition) and neutral videos. The figure shows the temporal fluctuation of mean heart rate. The dashed line between [−1] and [1] temporal window represents the fall of the sportsperson. The asterisk (*) denotes significant differences between temporal windows in empathy for pain (red).

To compare the temporal fluctuations in heart rate responses between the empathy for pain condition and the baseline condition, an ANOVA with the factors Condition (2) and Time Window (7) was conducted. The ANOVA found no significant interaction (*F*_6,204_ = 1.32, *p* = 0.25) in the heart rate. There was a main effect in the time window in the empathy for pain conditions (*F*_6,204_ = 4.79, *p* < 0.001). There was no significant main effect of emotional condition. As can be seen from Figure 4, empathy for pain conditions reported a significant decrease (*p* < 0.05) in the last two windows [3, 4] compared to the window before the fall [−1].

### 3.2 First-person result

This section presents the main findings of the phenomenological analysis conducted on the microphenomenological interviews following the participants’ viewing of fall videos. The analysis focused on the temporal dynamics of the experience. First, we present descriptions of the phenomenological dimensions that emerged in the analysis, followed by the full temporal structure of the empathy experience (a more comprehensive analysis of 28 out of the 35 participants, as well as a review of the codebook, can be found in our previous publication (Martinez-Pernía et al., 2023).

#### 3.2.1 Phenomenological categories

Following our analysis, four main phenomenological categories emerged (temporality, bodily resonance, kinesthetic motivation, and attentional focus). One category is a temporal category that persists throughout the entire empathic experience, evolving in synchrony with the events sportspersons undergo while engaging in extreme sports. The other three categories, which traverse temporality, are termed bodily resonance, kinesthetic motivation, and attentional focus. Below, we delve into these dimensions of the experience.

A consistent temporal structure was identified in the analysis and was observed across all participants. This temporal pattern exhibits fluctuations that occur in synchrony with the events experienced by the sportsperson. These fluctuations are framed within three distinct experiential phases: anticipatory, climax, and recovery. During the anticipation phase, which develops before the sportsperson falls, the participants intuitively perceive the forthcoming occurrence of an accident, marked by a gradual intensification of bodily sensations.

*“It’s going up, I feel a nerve… as if one were seeing it in real life… it’s a sensation, I could call it an instinct that it’s going to fall… it’s going to fall…”* (P20).

During the climax phase, when the sportsperson is about to fall and immediately during the fall, participants experienced the maximum intensity of bodily feelings. An excerpt from a participant’s interview is provided below.

*“I feel the tension as if it were contracting, releasing, here it enters my stomach; there I feel the, the negative. I also feel my whole body more, more alert in front of these videos. In the moments, of the fall itself, of when “paff” the person hits the ground, there I felt it stronger.”* (*P10*).

*Finally, in the recovery phase, which corresponds to the moments after the sportsperson’s fall, participants experienced a decreased intensity of bodily feelings*

*“…the sensation is brutally relieved when it already fell”* (*P12*).

In the analysis of synchronic categories within each temporal phase, three primary categories were identified: bodily resonance, kinesthetic motivation, and attentional focus.

The first category, called bodily resonance, captures the intimate, lived experience of participants as their bodies are affected by and moved by the actions of the sportspersons. For example:

*“I know a fall is coming, like first being expectant before the fall. I feel a little nauseous, anguish too, and my chest is tight. Being with this feeling of wanting to get away, like feeling that my body was going backward”.* (P10).

The second category, called kinesthetic motivation, captures the driving force experienced by individuals when witnessing the actions of sportspersons, prompting them to either safeguard themselves (protective motivation) or assist others (prosocial motivation).

An example of protective motivation is as follows:

“…*is the sensation of the body that you clench everything: your hands, arms, legs and back muscles tighten. It is like a feeling of protection, of how the body feels threatened by a predator, so to speak, that is the sensation of activating all the senses and tightening up”.* (*P26*).

An example of prosocial motivation is as follows:

*“My muscles were contracting more, I felt my knees buckling. I felt like, if I moved somehow, I was going to keep them from falling. I kind of engaged with them and felt that if I controlled my body, they wouldn’t fall”* (*P16*).

And finally, the third phenomenological category named attentional focus, reflects the very spots and instances that captivate and hold participants’ attention as they watch sportspersons in action during extreme sports. For example:

*“I immediately noticed that my body was very different… I felt my muscles contracting”* (*P20*).

#### 3.2.2 Experience structure

**Figure 5:**
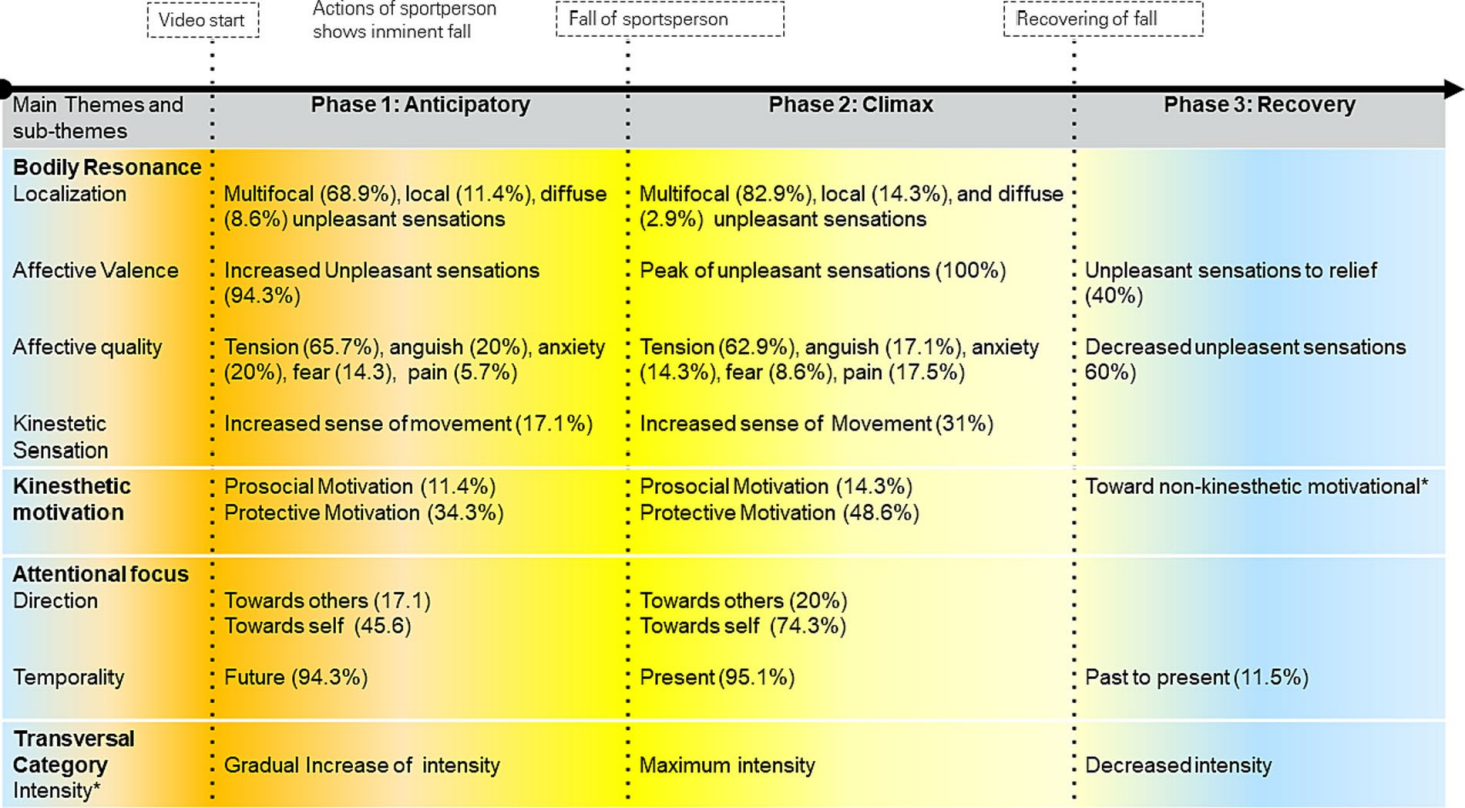
Temporal structure of empathy for pain. The arrow at the top of the figure indicates the time evolution of the experience. The dotted lines show the points of change from one phase to the next phase. Above the arrow are shown the video’s events concerning the participants in each phase. The cross intensity refers to the category that characterizes each phase. This category is integrated with the video moments perceived by the participants to define each phase. The asterisk (*) indicates an emergent category following refinement of the temporal structure, previously described as none. The color shows the temporal flow of intensity from gradual increase (orange), to climax (yellow), and then decrease (blue). The % represents the percentage of the total sample in the category.

The previously mentioned four main phenomenological categories (temporality, bodily resonance, kinesthetic motivation, and attentional focus) are strongly intertwined in an empathic experiential structure. For a complete visualization of the empathic structure and a more comprehensive breakdown, including the percentages of participants experiencing each aspect, please refer to Figure 5.

### 3.3 Anticipatory phase

During the anticipatory phase of bodily resonance, participants engaged in a profound bodily experience triggered by the physical actions of the sportspersons. This phase was primarily characterized by a keen sense of anticipation as participants intuitively grasped the unfolding events and directed their attention toward the future state of the sportsperson. Notably, a significant majority of participants described multifocal sensations, underscoring the widespread distribution of bodily resonance throughout their physical beings. The localization of these sensations were experienced in prominent bodily regions including the abdomen, chest, heart, face, and extremities. These sensations were intertwined with a subtle yet discernible undercurrent of negative affective valence, gradually intensifying as the experience transitioned toward the climactic phase. This negative valence was manifested through various affective qualities such as tension, pain, fear, anguish, and anxiety, with tension as the predominant emotional tone. Additionally, participants experienced subtle kinesthetic sensations, which manifested as sporadic instances of increased bodily movement and transient feelings of imbalance. Furthermore, they navigated the interplay between two distinct motivations—a self-protective motivation and a prosocial motivation. Participants’ self-protective motivation was marked by a sense of detachment while watching the video. It was as though participants’ bodies were preparing to distance themselves from the impending event. Simultaneously, their attention was drawn inwards, focusing on personal discomfort and sensations of rejection. In contrast, participants’ prosocial motivation was driven by a desire to prevent injury or a fall in the other person and their attention directed toward others. This outward attention encompassed feelings, actions, or thoughts directed at the suffering individuals, revealing the complexity of their responses during this phase of bodily resonance.

### 3.4 Climax phase

In the climax phase of bodily resonance, participants continued to undergo significant changes in their bodily experiences and emotional responses concerning the fall of the sportsperson. The participants’ attentional focus during this phase shifted toward a synchronized experience with the suffering of others in the present moment of fall. This shift from future anticipation marked a temporal synchronization of sensations concerning the observed pain, intensifying their connection to the sportsperson’s experience. Importantly, negative affective valence reached its peak intensity during this phase as participants experienced a heightened emotional response while observing the sportsperson’s pain. The localization of bodily sensations shifted to prominent areas such as the abdomen, chest, heart, face, and extremities, reflecting a different pattern from the anticipatory phase. Affective quality remained characterized by sensations of tension, but pain sensations became more pronounced.

Kinesthetic sensations, such as increased movement and feelings of bodily imbalance were more prominent during the climax phase, indicating a greater level of subjective engagement. This phase represented the zenith of bodily resonance, marked by heightened emotional intensity and kinesthetic involvement in response to the observed pain. Furthermore, during this phase, the experience of kinesthetic motivations was heightened. For instance, self-protective motivation remained prevalent, accompanied by sensations of heightened bodily tension and a sense of detachment from the other person’s suffering. Prosocial motivation persisted, reflecting the desire to prevent injury or a fall concerning the other person.

Interestingly, both in the preceding phase and the current one, participants identified two distinct experiences, termed self-centered empathy and other-centered empathy. Self-centered empathy is characterized by an attentional focus on one’s own experience, driven by a motivation for self-protection (N = 27). On the other hand, other-centered empathy involves directing attention toward the experience of the sportsperson’s pain and is accompanied by a motivation to offer assistance (*N* = 8).

### 3.5 Recovery phase

The recovery phase marked a transition in bodily resonance, reflecting a state of relief following the intense experiences of the climax phase. In this moment, participants’ attention was enveloped by a palpable sense of relief, and, some were attuned to the aftermath of the fall. During this phase, participants reported a decrease in the intensity of negative affect, signifying a release from the heightened emotional state observed in the climax phase. The affective quality transitioned toward sensations of relief as participants began to feel a tangible release from the preceding emotional intensity. In contrast to earlier phases, participants felt kinesthetic sensations and motivation diminish during the recovery phase. Overall, the recovery phase represented a shift toward emotional relief and a return to a more stable bodily state following the intense emotional and kinesthetic responses observed in the climax phase. Figure 5 shows the temporal structure of empathy for pain.

### 3.6 Bodyssence result

**Figure 6:**
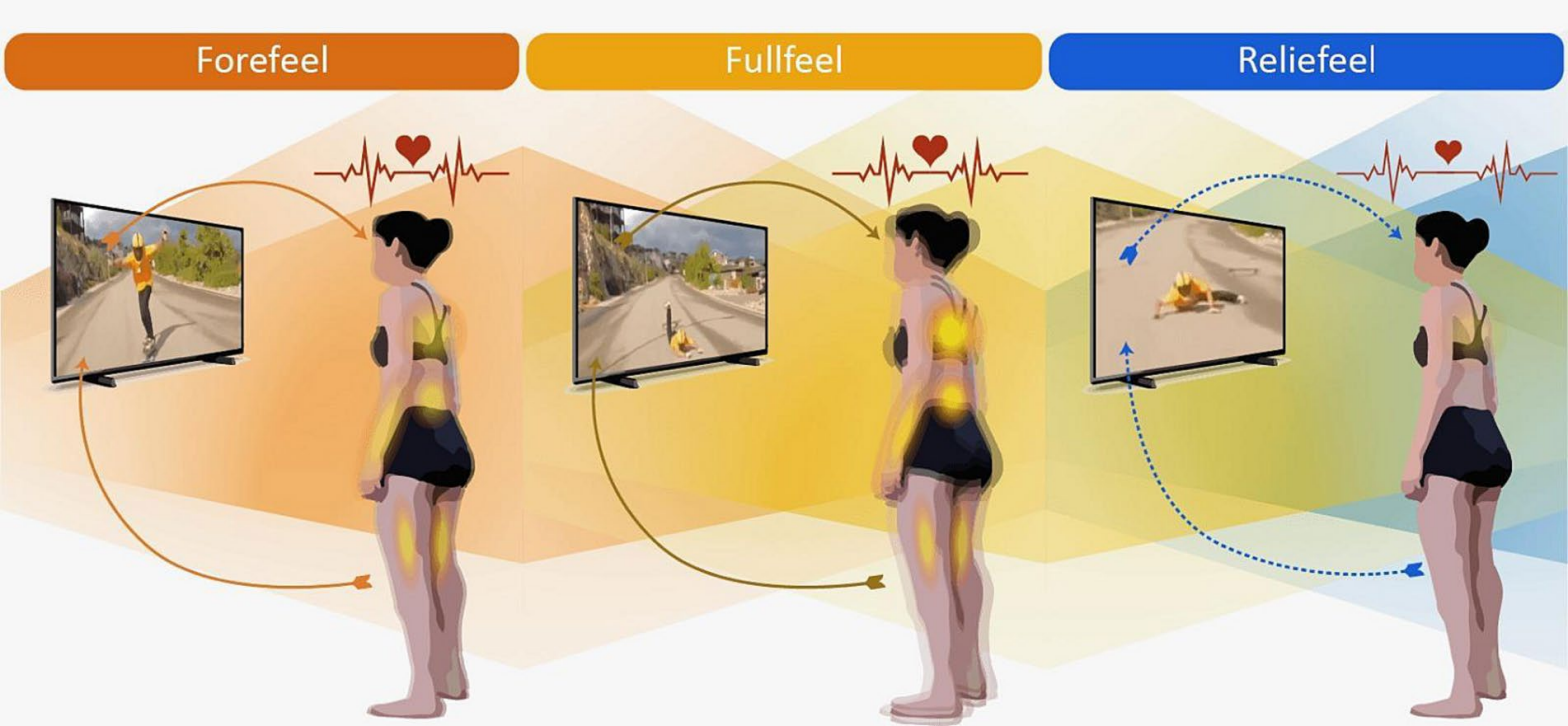
Illustration of Empathy Bodyssence. This figure depicts the interwoven phenomenological and neurophysiological phases of empathy for pain, graphically representing the seamless integration of first-person experiential data (1p) and third-person physiological data (3p). These distinct phases collectively illuminate the ‘bodyssence of empathy,’ showcasing the dynamic interplay between an individual’s physiological reactions and their concurrent lived experiences in response to observed events. Forefeel (orange hue), as the initial phase, embodies the body’s intuitive pre-reflective knowledge, with participants sensing the imminent physical accidents of extreme sportspersons, a state that is characterized by the most subdued levels of postural movement and elevated heart rate during the temporal dynamic of empathy. Fullfeel (yellow hue) represents the peak of empathetic connection, characterized by intense negative emotions, prominent bodily sensations, heightened kinesthetic sensations. This phase is distinguished by an increase in postural movement and decrease in heart rate in specific moments of the temporal dynamic of empathy. Reliefeel (blue hue): participants experienced a decrease in emotional intensity and a concurrent sense of relief, with postural control returning to an equilibrium and heart rate remaining low. Throughout all three phases, electrodermal activity was consistently elevated compared to the baseline condition.

After presenting the main results from physiological and phenomenological data, this section provides an integration of both dimensions. The phenomenological findings highlight three temporal phases: anticipatory, climax, and recovery. In the neurophysiological data, we identify three sequential temporal phases: the pre-fall period followed by the post-fall period, which is further divided into an early and a later response. Integrating both sets of data (1p and 3p) gives rise to three synchronously interwoven phases: the anticipatory temporal phase coinciding with the pre-fall period, the climax temporal phase aligning with the early post-fall response, and the recovery temporal phase paired with the later post-fall response. Thus, we illuminate the ‘bodyssence of empathy,’ a fusion of third-person neurophysiological data and first-person phenomenological insights. At its core, bodyssence exemplifies the rich interplay between our physiological responses and our deeply felt experiences coordinated with the events occurring in the environment (Figure 6). The three successive and distinctive phases of “empathy bodyssence” are listed and described as follows:

**Forefeel**: When extreme sportspersons began performing their acrobatics, participants experienced a pre-reflective felt knowledge or prediction of an accident. They experienced a gradual increase in the intensity of their negative emotions, multiple bodily sensations, low postural movement (AP-COP), and a high heart rate during the temporal dynamic of empathy. The longer participants watched the sportsperson, the more intense their experience. In this phase, participants also felt different kinesthesis motivations and focused attention. The increase in emotional intensity was accompanied by a high electrodermal activity compared to baseline condition.

**Fullfeel:** At some point during the sportsperson’s acrobatics, they lost their balance; the impact with the ground lasted a few brief seconds. In these moments, participants’ bodily resonance peaked. They were immersed in intense negative emotions, particularly in response to the sportsperson’s evident pain. This emotional surge was mirrored in bodily sensations, most prominently in the abdomen, chest, heart, face, and extremities, intertwined with kinesthetic sensations of movement and imbalance. As these feelings escalated, the amplitude of postural movement increased and reached its maximum point in seconds 2 and 3 post-fall, while the heart rate decreased in its last second, and the electrodermal activity remained consistently high.

**Reliefeel**: As the sportsperson was attempting to rise from the ground after their fall, there was a shift in the participants’ experiential state. Their previously heightened emotional intensity began to wane, mirrored by the physical relaxation they felt coursing through their bodies. This easing of negative emotions manifested in sensations of relief, tranquility, and diminished concern. Concurrently, as the participants watched the sportsperson try to recover, their postural stability began to be restored in the last second, evidenced by a reduction in the amplitude of their anteroposterior movements. This sensorimotor return to equilibrium was characterized by the sustained low heart rate. The electrodermal activity remained high compared to baseline condition.

## 4 Discussion

Our research endeavored to explore the multifaceted nature of empathy within an enactive framework. To facilitate this exploration, we introduce the novel concept of “bodyssence” (body + essence). This neologism encapsulates the holistic convergence of our neurophysiological reactions with our lived, subjective experiences. Our primary objective was to study how the temporal evolution of motor, physiological, and phenomenological facets shapes our empathetic consciousness in synchrony with the experience of the sportsperson enduring a physical accident. Our research on empathy bodyssence reveals a bodily experience marked by three consecutive phases named Forefeel, Fullfeel and Reliefeel. Bodyssence emphasizes the dynamic and temporal nature of these dimensions offering insights into how our embodied experiences evolve and resonate with both our internal states and external influences.

In the following paragraphs, we explore two significant concepts. These are the temporal dimension of bodyssence within the overall experience and the pivotal role that bodily resonance plays in fostering empathy for pain.

### 4.1 The temporality of bodyssence

The temporality of the bodyssence concerning the external referent of the fall suggests that there is a bodily attunement of the observer with the expressive behavior of the sportsperson shown in the video. Body attunement has been reported in several studies that show temporal coordination of individuals’ behaviors that manifest spontaneously in our daily interactions. For example, studies have identified that when observing the pain of another, there is a physiological and brain synchrony (Goldstein et al., 2017, 2018; Peled-Avron et al., 2018). In our study, the temporal coordination of bodyssence has been found in the three phases, Forefeel, Fullfeel, and Reliefeel.

In the Forefeel phase, the temporality of the embodiment reveals that when participants direct their attention toward an impending future fall, they experience multiple bodily sensations and show less postural sway. Similar to the findings of our study, previous studies have demonstrated that by perceiving the bodily cues of another, agents can predict the future actions of the another (Iacoboni et al., 2005). Likewise, the bodily cues of another being are perceived as a meaningful action and affective state through bodily resonance (Tanaka, 2015). Overall, the body’s feeling and movement response shows that the bodyssence is an active participant in the implicit knowledge of the future state of another (the oncoming fall).

In the Fullfeel Phase, the findings reveal that in the maximum intensity of the videos, the embodiment appears with a higher postural oscillation, bodily sensation, and unpleasant valence. This finding is consistent with the findings of studies previous that illustrated the responses of the sensory-motor cortex (Riečanský and Lamm, 2019), postural movement (Gea et al., 2014; Lelard et al., 2017, 2019), autonomic system (Eisenberg et al., 1988, 1991) and phenomenological insights (Fuchs, 2017) in the presence of others’ suffering. Despite the constant electrodermal activity in all phases, its elevation in the empathy for pain condition indicates autonomic embodied resonance to others’ pain, consistent with previous studies (Hein et al., 2011; Lelard et al., 2014).

In addition, subjects observing the fall report a set of affective qualities and different bodily sensations in different parts of the body. This reflects that a bodily resonance, not only occurs in objective bodily phenomena but can also be accessed from subjectivity. Bodily resonance also includes the tendency to action or kinesthetic motivation, which do not necessarily manifest themselves in physical space, but are phenomena that manifest themselves in lived space (Fuchs, 2017).

In the Reliefeel Phase, temporal attunement has also been revealed with the decrease of intensity of the bodily feeling experience and the decrease of postural sway and heart rate (Lang et al., 1993; Lelard et al., 2014, 2017; Mouras and Lelard, 2021). While some studies interpret the reduction in postural sway and heart rate after exposure to highly aversive film content, such as scenes depicting car accidents and dead bodies, as indicative of a freezing-like response (Hagenaars et al., 2014), another proposal suggests that rapid deceleration in heart rate following a stressful situation allows a recovery state in the absence of imminent danger (Bernston et al., 1993). This implies, in conjunction with phenomenological data, that the decline in heart rate may be linked to a diminution of anguish following the peak experience of witnessing someone else fall.

Interestingly, the heart rate demonstrates swift adaptation and decreases, while electrodermal activity remains relatively unchanged. These findings suggest that, even though sympathetic system activation persists, as indicated by elevated electrodermal activity, subtle changes such as an increase in the parasympathetic system or a decrease in the sympathetic system (e.g., Weissman and Mendes, 2021) could signify an augmented state of relaxation following the observation of the fall. Additionally, the temporal invariability of the electrodermal activity aligns with previous reports indicating a slow temporal adaptation of electrodermal activity to exposure to aversive stimulus (Lelard et al., 2014).

The depiction of these phases reveals a noteworthy alignment between subjective experiences and physiological data, which has been a subject of controversy in some studies on empathy for pain. For instance, a study shown that mental simulation had a modulatory effect on postural sway but not on self-reported measures of empathy (Beaumont et al., 2021). While the authors debate the underlying physiological mechanisms contributing to the absence of results in self-reporting, it seems that enhancing subjective data collection could yield more detailed insights about the findings (Martínez-Pernía et al., 2021; Vergara et al., 2022). Our study underscores the importance of deepening subjective experiences to accurately grasp how individuals experience specific points of exposure to the video. This allows us to make a more detailed interpretation of the physiological findings. Thus, future research adopting traditional paradigms could benefit from incorporating phenomenological interviews or even simpler phenomenological self-reports, as previously utilized in research (Grice-Jackson et al., 2017a,b).

### 4.2 Beyond freezing and fight or flight actions: the body as a source of primary empathy

Our findings concur with a multitude of studies emphasizing the pivotal role the body plays in empathetic responses, particularly in the context of pain. For instance, recent research has highlighted that specific neural regions activated during first-hand pain experiences are similarly triggered when observing the pain of others (Riečanský and Lamm, 2019). Specifically, when individuals witness somatic pain, such as viewing others in painful situations, there occurs a pronounced activation in regions associated with negative emotional processing and in areas related to somatosensation and skeletal muscle control (Riečanský and Lamm, 2019). Studies suggest that emotion recognition becomes compromised when facial mimicry is restricted, whether through the impossibility of using certain muscles (e.g., biting a pen while observing others’ emotions) (Oberman et al., 2007; Borgomaneri et al., 2020) or muscle immobilization via botox injection (Neal and Chartrand, 2011), as well as when bodily movement is restricted (Reed et al., 2020). Collectively, this body of evidence underscores the indispensable role of bodily experiences in comprehending the emotions of others.

Considering the role of the body in social cognition, objective bodily responses and subjective bodily descriptions go beyond the consideration of mere freezing or flight responses to an aversive stimulus (Azevedo et al., 2005; Hagenaars et al., 2014). Rather, we suggest that bodily experience has an interpersonal function to allow for resonance with and understanding of the other person. This means that the other’s bodily expression appears to us as meaningful and affective actions that express their intentions, needs, and goals in a shared context (Gallagher, 2012; Tanaka, 2015; Fuchs, 2017). Those expressions affect us and are experienced through and with our bodies (Cea and Martínez-Pernía, 2023), which defines the concept of bodily resonance. Fuchs (2013, 2017) has proposed two components of bodily resonance: an affective dimension (the body is affected by events through bodily sensations) and an e-motive dimension (the body tends to act through body movement). Both components are related to our findings that show an affective quality of bodily sensations, kinesthetic sensations and motivation, and changes in postural sway.

Within the emotional dimension, participants expressed dual motivations: to protect themselves and/or to adopt the perspective of the person experiencing distress and assist the person suffering the fall. These intentions highlight the active facet of bodily resonance and suggest that it also modifies bodily sensations in a nuanced sensorimotor cycle. Intriguingly, past research has recognized both aversion-motivated and approach-motivated states. In our findings, we discerned two distinct experiences among participants—those driven by self-oriented protective actions and those motivated by other-oriented actions. Accompanying these intentions to act were differing attentional foci—some directed inwardly and others directed outwardly. Both dimensions underscore the intricacies of these phenomena (for more details, see Martinez-Pernía et al., 2023) that previous studies, which largely relied on physiological reports and self-reporting, might not have fully captured. Further research could explore the relationship between both experiences and physiological responses. For this purpose, a special focus might be needed to increase the sample size and conduct robust between-group comparisons. For example, our study lacks sufficient statistical power to differentiate between these two experiences, given the unbalanced sample sizes in each group (27 self-protective vs. 8 prosocial). Incorporating such insights into subsequent investigations will undoubtedly contribute to a more comprehensive understanding of these relationships.

In another vein, the results of this study show that bodily sensations were described in a multifocal manner during the climax in various parts of the body, encompassing the level of the chest and/or extremities. No subject described a general pain sensation or an absence of bodily sensation. This contrasts with what was found by Grice-Jackson et al. (2017a), who found three different experiential structures in a classical picture-based paradigm: non-responder, sensory/localizer, and affective general. We suggest that given the intensity and type of stimulus, our experiential structures cluster into multifocal responders.

### 4.3 Limitations of the study

One of the limitations of the study is the absence of an ecological stimulus that occurs in natural contexts. Therefore, the conclusions raised in this article are limited to the laboratory context. The authors propose to change the classical neuroscience paradigm to paradigms that have a greater natural context (i.e., with multisensory contexts, free movement, and real interaction with others; Troncoso et al., 2023). However, our study advances the ecology by allowing participants to move freely in a bipedal position. The use of the instruments enabled us to evaluate embodiment phenomena by allowing free movement in a natural bipedal position. It is suggested that future empathy studies use paradigms in natural contexts and instruments that allow the evaluation of neural correlates at the same time (e.g., mobile eeg).

Another limitation is that we queried subjects about the impact of the maximum intensity video, which was different for everyone and could generate intragroup differences in the type of behavior. However, in the interviews, the subjects explicitly stated that they presented a similar experience in the other videos. For future studies, we suggest using the same scenes for all participants and integrating the same physiologically evaluated scenes.

Another limitation of this study pertains to the baseline condition video, which inherently differs from the videos showing the falls. Traditionally, the empathy for pain paradigm utilizes more analogous images—one showing pain and the other not showing pain. However, in our study, the baseline condition was specifically designed to gauge a fundamental neurophysiological and postural response. This provides a foundational reference allowing us to effectively compare the heightened responses evoked by the empathy for pain condition. However, an important advantage of our study is the integration of the phenomenological approach. While our stimulus may not be a direct control condition in comparison to the empathy for pain video, our experimental design rooted in phenomenology allows us to delve deeply into the moment-to-moment lived experiences of participants. By doing so, we not only understand the genuine reactions of participants but also grasp the intricate dynamics of empathy as experienced in real-world conditions. For future research, we suggest considering the use of comparable videos instead of videos with different content to enhance experimental control. This would ensure that participants’ responses are more comparable and that differences in stimulus nature do not become a source of confusion. Improving the uniformity of stimuli can contribute to greater robustness and generalizability of findings.

Additionally, expanding the research scope could involve extending physiological data collection before and after stimuli exposure, along with deepening phenomenological interviews. This would offer a comprehensive understanding of physiological responses, clarifying baseline patterns and addressing gaps like those seen in electrodermal activity analysis. Additionally, exploring anticipatory physiological changes before stimuli exposure and post-stimulus recovery could unveil preparatory mechanisms and adaptive responses. Such an approach is especially promising for dynamic stimuli studies, offering more insights into temporal dynamics.

### 4.4 Constraints on generalizability

The sample was confined to a segment of young, educated, and healthy individuals, which may not be representative of the broader population. It is also crucial to consider potential cultural and contextual factors that could influence the phenomenological and physiological aspects of empathy. For instance, individuals consistently exposed to pain situations, such as health professionals (Decety et al., 2010), and those displaying lower levels of empathic resonance, like individuals with psychopathy (Decety et al., 2013), may find it worthwhile to replicate this study. Understanding how individuals from different backgrounds perceive the suffering of others could offer valuable insights into the interplay between experience-physiology and context.

## 5 Conclusion

Our results reveal a temporal structure of the bodily experience and physiology of empathy for pain, marked by three consecutive phases that synchronize with the evolving experience of the sportsperson. This highlights how the body resonates dynamically, both in its subjective realm and physiological realm, when experiencing the suffering of another. Furthermore, the current enactive framework illustrates how the mutual interaction between phenomenological data and physiological data allows them to inform each other with higher precision to understand the empathy bodyssence.

This research not only validates the depth and applicability of the enactive approach but also encourages a collaborative effort among philosophers and neuroscientists, and associated disciplines. The development of empirical evidence within the enactive framework could not only refine the theoretical propositions but also, and only in this way, ensure the paradigm’s validity and enduring significance, thereby paving the way for its lasting legacy.

## Data availability statement

The datasets and analysis presented in this study can be found in online OSF repository: https://osf.io/dtcr2/.

## Ethics statement

The studies involving humans were approved by the “Scientific Ethics Committee of the Servicio de Salud Metropolitano Oriente” and the “Research in Humans being Ethics Committee of the Medicine Faculty, Universidad de Chile.” The studies were conducted in accordance with the local legislation and institutional requirements. The participants provided their written informed consent to participate in this study. Written informed consent was obtained from the individual(s) for the publication of any potentially identifiable images or data included in this article.

## Author contributions

AT: Conceptualization, Data curation, Formal analysis, Investigation, Methodology, Visualization, Writing – original draft, Writing – review & editing. DM-P: Conceptualization, Formal analysis, Funding acquisition, Investigation, Methodology, Project administration, Resources, Writing – original draft, Writing – review & editing. KB: Data curation, Formal analysis, Methodology, Writing – review & editing. ÁR-R: Data curation, Formal analysis, Methodology, Writing – review & editing.

## Footnotes

^1^Twenty-eight participants were extracted from our previous study (Martinez-Pernía et al., 2023)). In our previous study we focused on exploring the structure of experiences (self-centered and other-centered empathy) based on non-temporal phenomenological analysis.
